# Correlates of Orthographic Learning in Swedish Children With Cochlear Implants

**DOI:** 10.3389/fpsyg.2019.00143

**Published:** 2019-03-01

**Authors:** Malin Wass, Ulrika Löfkvist, Lena Anmyr, Eva Karltorp, Elisabet Östlund, Björn Lyxell

**Affiliations:** ^1^Department of Business Administration, Technology and Social Sciences, Luleå University of Technology, Luleå, Sweden; ^2^Department of Special Needs Education, University of Oslo, Oslo, Norway; ^3^Department of Clinical Science, Intervention and Technology, Karolinska Institutet, Solna, Sweden; ^4^Department of Social Work in Health, Karolinska University Hospital, Stockholm, Sweden; ^5^Department of Otorhinolaryngology, Karolinska University Hospital, Stockholm, Sweden; ^6^Department of Behavioral Sciences and Learning, Linköping University, Linköping, Sweden

**Keywords:** orthographic learning, reading fluency, deaf and hard of hearing children, cochlear implants, reading development

## Abstract

This study set out to explore the cognitive and linguistic correlates of orthographic learning in a group of 32 deaf and hard of hearing children with cochlear implants, to better understand the factors that affect the development of fluent reading in these children. To date, the research about the mechanisms of reading fluency and orthographic learning in this population is scarce. The children were between 6:0 and 10:11 years of age and used oral language as their primary mode of communication. They were assessed on orthographic learning, reading fluency and a range of cognitive and linguistic skills including working memory measures, word retrieval and paired associate learning. The results were analyzed in a set of correlation analyses. In line with previous findings from children with typical hearing, orthographic learning was strongly correlated with phonological decoding, receptive vocabulary, phonological skills, verbal-verbal paired-associate learning and word retrieval. The results of this study suggest that orthographic learning in children with CI is strongly dependent on similar cognitive and linguistic skills as in typically hearing peers. Efforts should thus be made to support phonological decoding skill, vocabulary, and phonological skills in this population.

## Introduction

### Language, Cognition and Reading Skills in Deaf and Hard of Hearing Children

For deaf and hard of hearing children who are fitted with cochlear implants, here referred to as *children with CI*, the auditory signal is degraded and has poorer frequency resolution compared to typical hearing (Pisoni et al., 2008; Brown and Bacon, 2010). Thus, their perception of the acoustic-phonetic details of language is poorer compared to that of individuals with typical hearing (TH) (Pisoni et al., 2008; Brown and Bacon, 2010; Hall and Bavelier, 2010) and this, in turn, leads to underspecified neural representations of speech and poorer phonological skills (Lyxell et al., 2008; Pisoni et al., 2008; Geers and Hayes, 2011). In the current paper, the broad term phonological skills, is used to denote the awareness of and sensitivity to the sound structure of language (e.g., Anthony and Francis, 2005; Anthony et al., 2002). Phonological skills may be subcategorized into a number of subskills including the ability to discriminate, store and manipulate speech sounds. Because oral language and phonological skills are strongly associated with the development of reading ability (e.g., Ehri, 2005), children with CI face more challenging conditions for learning to read than children with TH (Conway et al., 2011; Nakeva von Mentzer et al., 2014). In a number of studies, children with CI have been found to have poorer reading skills compared to typically hearing comparison groups (e.g., Geers, 2003; Harris and Terlektsi, 2010; Johnson and Goswami, 2010; Geers and Hayes, 2011). In a longitudinal study by Geers and Hayes (2011), children with CI were assessed on measures of reading in elementary grades, at age 8–9, and then again in high school, at age 15–18. The results showed that most of the students had a similar performance, as compared to norms for hearing children, across both measurements. However, 28% of the children showed a relative drop in performance between elementary grades and high school on the Word Attack subtest of the Woodcock Reading Mastery Test, which measures phonological decoding. Similar findings of a relative drop in reading performance with increasing age has been reported for children with hearing loss who use traditional hearing aids (Marschark and Harris, 1996).

Although the relative performance of children with CI on different aspects of reading has rarely been directly addressed in previous research, some findings would suggest relatively more difficulties with phonological decoding processes than with orthographic word recognition (e.g., Johnson and Goswami, 2010; Nakeva von Mentzer et al., 2014). The present study investigated the ability to memorize the spelling of new written words – *orthographic learning* – in profoundly deaf children with CI.

### Reading Development in Children With Typical Hearing

Recent cross-linguistic studies have demonstrated that the most important predictors of early reading development are a child’s phonological skills including letter knowledge and phonemic awareness, but also verbal fluency (e.g., Caravolas et al., 2012, 2013; Moll et al., 2014). Phonemic awareness is an aspect of phonological skill, which refers to the knowledge of how to distinguish the separate phonemes in pronunciations of words (Ehri, 2005). Verbal fluency refers to the ability to quickly retrieve verbal labels, often measured in rapid naming tasks.

Phonological skills are particularly important for learning to sound out words letter by letter in a process referred to as *phonological decoding* (e.g., Ehri, 2005; Melby-Lervåg et al., 2012) whereas fluency measures are stronger predictors of reading speed, in particular for languages with less consistent orthography, such as English (Moll et al., 2014).

Beginning readers typically read mainly by means of phonological decoding but as they become more experienced they gradually learn to recognize whole words by sight (Coltheart et al., 2001; Ehri, 2005). This quick and automatized reading process is known as *orthographic word recognition* (Coltheart et al., 2001) and it is achieved through comparing the visual characteristics of incoming words to long-term memory representations of their spellings (Castles et al., 2007, 2009).

In order to read fluently by means of orthographic word recognition, children need to build up a large lexicon of orthographic representations in their long-term memory. The new orthographic representations are acquired through a process referred to as *orthographic learning* (Share, 1995) in which the child memorizes the spelling and visual characteristics of written words as they read independently by means of phonological decoding (Share, 1999, 2004). Efficient orthographic learning is thus necessary in order to become a fluent reader.

Orthographic learning is typically measured in tasks in which children are presented with new nonsense words (Swedish example ‘*rovna*’) of which they are asked to memorize the spelling. The nonsense words may be presented either in a semantic context (short stories) or without semantic context, for example as single words in tables. After the presentation phase, orthographic learning is typically assessed in three types of tasks: (1) in tasks where reading speed for the target non-words is measured and compared to the reading of homophones of the targets, (2) in spelling tasks, and (3) in recognition tasks where the word should be recognized from amongst a number of phonological and/or visual distracter words.

### Predictors of Orthographic Learning

Phonological decoding has proved to be the most important predictor of orthographic learning in typically hearing children. This is, for example, demonstrated by decreased orthographic learning in studies designed to prevent phonological decoding, e.g., by simultaneously producing an irrelevant pseudo word at the time when they are exposed with the target non-word (e.g., Share, 1999, 2004; De Jong et al., 2009). According to the self-teaching hypothesis (Share, 1999), the active mechanism behind this relationship is that children memorize the spelling pattern of written words as they read by means of phonological decoding. Phonological decoding has also been found to be strongly associated with orthographic learning in deaf or hard of hearing Australian 9-year-old children using traditional hearing aids or CI (Wass et al., 2018). In TH children, orthographic learning has, besides phonological decoding, also has been found to be predicted by orthographic skills, i.e., knowledge about spelling and spelling rules (Cunningham, 2006; Conners et al., 2011) and paired associate learning (Wang et al., 2017). The concept *paired-associate learning (PAL)* is used to denote the ability to associate two pieces of information (Litt and Nation, 2014). The information may be of the same or different modalities, for example, two pieces of visual information (e.g., shapes), two pieces phonological information (e.g., words or non-words) or cross-modal association with one piece of phonological and one piece of visual information (e.g., shape and non-word). Paired-associate learning is used by young children when learning to name objects, and when learning the names and sounds of letters in the alphabet (e.g., Lervåg et al., 2009). It may also be involved in the acquisition of an orthographic lexicon later in reading development when children learn to associate whole words with their pronunciations without relying on phonological decoding (Hulme et al., 2007; Lervåg et al., 2009; Litt and Nation, 2014). This association process is most likely involved when learning the pronunciation of irregular words, which may not be correctly read by means of phonological decoding (e.g., Castles et al., 2007). It is further assumed that the association process, to a certain extent, occurs very early in reading development parallel to and probably even mediated by phonological decoding (e.g., Share, 2004). Paired associate learning has been found to be associated with orthographic learning in English speaking deaf and hard of hearing children (Wass et al., 2018).

*Receptive and expressive vocabulary*, respectively, are measures of lexical-semantic knowledge, which reflect a child’s comprehension of spoken words. Vocabulary is known to be important for most aspects of language and communication including reading acquisition (Baddeley, 2003) and orthographic learning (Ricketts et al., 2007; Ouellette and Fraser, 2009). Vocabulary has been reported to be more strongly related to word reading for children with hearing loss, than for TH children (James et al., 2008; Kyle et al., 2016). Vocabulary has also been found to be related to orthographic learning in TH children (Ricketts et al., 2007; Ouellette and Fraser, 2009) and in children with moderate to profound hearing loss (Wass et al., 2018). According to Ehri (2014), an extensive spoken vocabulary facilitates orthographic learning by means of self-teaching because it may help children in the process of matching written words, that the children have not seen in print before, to their spoken form in long-term memory. Recent findings from an Australian study by Wegener et al. (2018) further suggest that for TH children who have already acquired basic reading skill, the process of orthographic learning may be initiated in parallel with vocabulary acquisition. That is, when children hear a spoken word for the first time, they may start to build up an anticipated orthographic representation of the word based on its phonological form. These anticipated orthographic representations are suggested to facilitate reading later, when the word is seen in print for the first time (Wegener et al., 2018).

*Phonological short-term memory* refers to the short-term storage of phonological information, and articulatory rehearsal of phonological/verbal information in order to refresh the memory trace (Baddeley, 2003, 2012). According to the multi-component model of working memory, these processes occur in the phonological loop component of working memory and are typically measured in tests of immediate recall of digits, words or non-words (e.g., Gathercole et al., 2004). In children with typical development, phonological short-term memory is important for vocabulary learning, both in the native language and in foreign languages (Baddeley, 2003, 2010; Gathercole, 2006; Repovš, 2006). Relationships between phonological short-term memory and reading skill have been demonstrated and in particular, measures of non-word repetition have been found to predict decoding skills and reading disability in children (Bishop, 2001; Bishop et al., 2004).

*Visuo-spatial short-term memory (VSTM)*: According to the multi-component model of working memory, VSTM processes involve storage and manipulation of visual and spatial information and occur in the *visuospatial sketchpad* component of working memory (Baddeley, 2003, 2010, 2012; Repovš, 2006). Research on the possible relationship between VSTM and reading skill is sparse and inconclusive and this may partly be due to the fact that different studies have tested different aspects of VSTM. For instance the visual information to be recalled may be presented in series as compared to simultaneously (c.f., Wang and Gathercole, 2013). Menghini et al. (2011) used a task to assess the ability to remember sequences of abstract visual figures and spatial positions (i.e., sequential visual working memory) in 8–13 years old children with dyslexia. The authors found that this group of children performed more poorly than a control group of typical readers on this task.

Gathercole et al. (2006) on the other hand investigated VSTM in children with reading disabilities in a test with simultaneous stimulus presentation of visual test items. The children with reading disabilities performed more poorly than a control group of children with typical reading development although the VSTM measure did not correlate with reading skill in this group.

Furthermore, Holmes et al. (2008), studied VSTM in adults with spelling difficulties. The authors found that the poor spellers were able to reproduce the order of symbol sequences equally well as a control group of good spellers, regardless of whether the symbols were presented simultaneously or sequentially.

In several studies, children with CI have been found to perform within the normal range, not significantly different from TH peers on measures of VSTM, both when the test stimuli has been presented simultaneously (e.g., Wass et al., 2008; Nakeva von Mentzer et al., 2014) and sequentially (Johnson and Goswami, 2010). Furthermore, Johnson and Goswami (2010) found that sequential VSTM accounted for unique variance in orthographic knowledge as measured by a word chains test where the children were asked to mark the boundaries between words, e.g., catsonglight should be cat, song and light. The authors speculate that the VSTM skills of the children support the development of orthographic knowledge when it comes to memorizing visual orthographic representations.

*Word Fluency* refers to processes that require strategic search and retrieval of words and concepts from long-term memory (e.g., Riva et al., 2000; Sauzeon et al., 2004). Word fluency is typically assessed in tasks where the participant is required to quickly retrieve words from long-term memory according to certain rules. The fluency measures are therefore categorized differently depending on the rules that are used for guiding retrieval. For example tasks that require the retrieval of words of a certain semantic category such, as animals, are referred to as *semantic fluency* and whereas the retrieval of words starting with a certain speech sounds such as f, a, or s is referred to as *phonemic fluency* (e.g., Troyer, 2000). Word fluency measures are related to lexical access and vocabulary in that they require quick retrieval of phonological and semantic information from long-term memory (e.g., Prigatano and Gray, 2008; Luo et al., 2010).

### The Current Study

We set out to investigate the cognitive and linguistic correlates of orthographic learning in a sample of Swedish children with CI. We also wanted to compare the pattern of correlations between orthographic learning and cognitive/linguistic skills to the pattern of correlations between word decoding fluency and cognitive/linguistic skills in order to find out whether orthographic learning is dependent on the same underlying processes as word decoding.

It is important to increase our knowledge about orthographic learning in children with CI as it has the potential to give insights into the process of reading acquisition in this population of children and possibly understand the reported relative drop in reading performance when the children grow older (c.f., Geers and Hayes, 2011).

This study was conducted as a part of a longitudinal project on reading development in children with CI. The children were assessed on a range of cognitive and linguistic measures, which have all been found to predict reading skill or orthographic learning in children with typical reading development. The children’s performance on measures of reading, cognition and language was also compared to age-norms or previously collected results from age-matched TH children.

This research was approved by the Research Ethics Review Committee at Linköping University (Dnr 2011/295-31).

## Methods

### Participants

Thirty-two children with cochlear implants participated in the study. Written informed consent was obtained from the children’s parents. All of the participants were implanted at the cochlear implant clinic, Karolinska University hospital where they were also followed up regularly once per year. Between those follow up appointments, the children attended some regular speech and listening rehabilitation at their local hospitals. It should be noted that the cochlear implant clinic at Karolinska University hospital has the highest number of cochlear implant patients in Sweden with a catchment area of approximately 5 million people. The sample was thus relatively representative of Swedish children with CI although the inclusion criterion was that the children should be able to follow the national school curriculum. The results from one of the children were excluded in all analyses because of a low score on non-verbal IQ (10th percentile on Raven CPM). Seventeen of the children were girls and 15 boys and the chronological age range was 6;0 – 10;11 years (mean 8;4 years). The sample was heterogeneous in terms of cause of deafness and age of implantation for first and second CI. Etiology and age at implantation for the sample is summarized in Table 1. None of the children had language levels on par with typically hearing peers before implantation except for the four children with deafness caused by meningitis. Those children were implanted at between 7 and 16 months of age. They were all diagnosed with meningitis (and consequently deafness) at 4+/-2 weeks before their age at implantation. These children were included in the current sample because they were deafened before 18 months of age, which denotes the start of a rapid development of vocabulary (review by Hoff, 2009) and they could thus be said to be prelingually deaf to a substantial extent. Furthermore, the possible advantage from early exposure to spoken language that these children may have had was also expected to be canceled out because normally hearing children with a history of meningitis have been reported to be in a less favorable position for language development compared controls with no history of meningitis (Anderson et al., 1997; Pentland et al., 2000; El-Kashlan et al., 2003).

**Table 1:** Etiology and age at implantation.

	Mean	SD (range)
Age at CI1 (months)	25.9	18 (7–69)
Age at CI2 (months)	31	23 (8–105)

	**# of children**	**Proportion**

Unilateral CI	6	6/32
Bilateral CI	26	26/32
**Etiology**		
**Acquired**		9/32
Congenital CMV	5	
Meningitis	4	
**Genetic**		14/32
Unspecific heredity^∗^	3	
Connexin 26	4	
Usher Type 1	2	
Jerve-Lange Nielsen syndrome	3	
Pendred’s syndrome	2	
**Unknown**	9	9/32


*^∗^Close family members also have a hearing impairment.*

The whole group of children were fitted with their first cochlear implant at 25.9 months on average (SD = 18; range: 7 months – 69 months). Twenty-six of the children (81%) had bilateral CI:s and were implanted with their second CI at 31 months on average (SD: 23; range: 8 months – 105 months). Twenty-seven of the children used oral language as their only mode of communication and 5 children used both oral language and sign language.

Speech perception in quiet was, as measured by phonetically balanced lists, on average 69.8% (SD: 15.3; range: 44–100). Speech perception data was missing for 3 of the children. Twenty-seven of the children were integrated in schools for TH children, 12 of those children had a teaching assistant to support them in class and they other 15 integrated children did not receive any support. Five children attended classes for children with hearing loss but with teaching in oral language, and 2 children attended sign language classes with teaching in both sign language and oral language.

The participants had percentile scores ranging between 25–95 (median: 75) on the Raven Colored Progressive Matrices test (Raven et al., 2003).

### Test Measures

A range of measures to assess reading ability, orthographic learning, paired-associate learning, working memory, and language skills were administered to all participants. An overview of the test measures is presented in Table 2.

**Table 2:** Tests administered together with proportions of children who performed within 1 standard deviation of the mean for their age, and means, standard deviations and range per test for the whole group of participating children.

Measures of language and cognitive skills	Test	Quantification	Proportion of children within or above 1 SD of TH age mean	Mean (SD)	Range
Complex working memory	Sentence completion and recall (Wass et al., 2008)	Number of correctly recalled words (maximum score = 18)	18/32	9.7 (4.3)	1–17
Visual short-term memory	Matrix Pattern Test (Wass et al., 2008)	Number of cells in the most difficult pattern correctly reproduced (maximum score = 8)	15/32	3.8 (1.5)	1–6
Phonological short-term memory	Non-word repetition (Wass et al., 2008)	Percent correctly reproduced consonants in whole test (100% = 120 consonants)	3/22	58 (18)	15–89
Visual–Visual PAL	Test adapted from Hulme et al. (2007)	Number of correct answers (maximum score = 20)	N/A	15 (4.4)	7–20
Visual-Verbal PAL	Test adapted from Hulme et al. (2007)	Number of correct answers (maximum score = 20)	N/A	6.7 (5.0)	0–18
Verbal-Verbal PAL	Test adapted from Hulme et al. (2007)	Number of correct answers (maximum score = 20)	N/A	2.5 (3.7)	0–15
Receptive Vocabulary	PPVT-III (Dunn and Dunn, 2007)	Number of correctly identified pictures (maximum score = 228)	25/32	114.4 (31.5)	51–176
Word Fluency	FAS (Benton and Hamshere, 1976)	Number of words starting with F, S, S produced within 1 min	28/31	15.5 (7.3)	2–30
	Animals (Benton and Hamshere, 1976)	Number of animals produced within 1 min	31/31	14.9 (4.0)	9–23
Expressive vocabulary	Boston Naming Test (Kaplan et al., 1983)	Number of correctly named pictures (maximum score = 60)	22/32	32.9 (10.7)	7–48
Phonological Skills	Phoneme deletion (Magnusson and Nauclér, 1993)	Number of correctly manipulated words (maximum score = 12)	N/A	9.3 (3.8)	4–12
Non-word decoding fluency	LäSt (Elwér, Fridolfsson, Samuelsson, and Wiklund, C. (2009)	Number of correctly read non-words in 2^∗^45 s (maximum score = 126)	31/32	48.2 (23.0)	11–98
Word decoding fluency	LäSt (Elwér et al., 2009)	Number of correctly read words in 2^∗^45s (maximum score = 200)	31/32	84.0 (38.9)	16–147
Orthographic skills	Orthographic Choices (Byrne et al., 2008)	Number of correctly identified spellings (maximum score = 40)	19/25	30.3 (9.3)	10–40
Orthographic Learning	Test adapted from Byrne et al. (2008)	Number of correctly spelled non-words (maximum score = 16)	16/32	6.31 (4.3)	0–15


*N/A in the column *proportions of children within or above 1 SD of TH age mean* denotes those test measures for which we did not have age norms or comparison data from children with typical hearing.*

#### Reading Measures

*Reading fluency* was assessed using the Swedish decoding test LäSt (Elwér et al., 2009). This test comprises two subtests: one using words and the other using non-words. In each subtest, the child was required to read correctly as many items as possible in 2^∗^45 s. The children received one credit for every item read correctly.

*Orthographic learning* was measured in a test adapted from the Scandinavian version of the Orthographic Learning test in Byrne et al. (2008). The target words in this test are identical to those in Byrne et al. (2008). In the present study, however, the target items were presented without a sentence context, similar to the procedure used by Nation et al. (2007). This adaptation was done in order to reduce test times for the children and make the test less taxing for children with relatively poor reading skills. It should be mentioned here that significant orthographic learning occurs irrespective of whether the words to be learned are presented with or without semantic context (Nation et al., 2007; Wang et al., 2011, 2017). The children were presented with words written on cards. Six word cards were presented at a time. Three of the cards had the same new non-word written on them and the other three had familiar words with the same number of letters, for example faus, korv, faus fisk, katt, faus. The child was asked to read the word on each of the cards and to try to recall the spelling of the new non-word, in this example faus. Incorrect pronunciations of target non-words were corrected by the examiner. After the presentation of three sets of cards, the child was asked to write down the three new non-words on a separate paper. This procedure was used for fifteen sets of cards (fifteen new non-words). The children’s spellings of each of the non-words were scored binary and they only received credits for completely correct spellings.

The Scandinavian version of the *Orthographic Choice test* used by Byrne et al. (2008) was used to measure orthographic skill. In this test, the child was presented with two alternative spellings of words and the task was to decide which spelling was correct. Maximum score in this test is 40.

#### Measures of Language and Cognitive Skills

##### Paired associate learning (PAL)

*Paired associate learning (PAL)* was assessed in a Swedish version of the test used by Wang et al. (2017) and Wass et al. (2018). Three types of PAL were tested: visual – visual; visual – verbal, and verbal – verbal.

##### Verbal – verbal PAL

In this task, the child was asked to learn pairs of spoken nonsense words using two sets of four CVC nonsense words (vak, dap, lut, hab; jom, neg, tem, and pog). The child was first asked tp repeat the nonsense words and then the experimenter said the associations twice, for example, ‘dap’ goes with tem’, [2-s interval], ‘dap goes with tem’. After all pairs were introduced, the child was asked ‘which other word goes with dap?’ The responses were recorded by the examiner and feedback of the same kind as in the initial presentation was provided, for example, “Do you remember? dap goes with tem”. The procedure was repeated five times, making a total of 20 learning trials/responses.

##### Visual – verbal PAL

In this task, the child was asked to learn shape – nonsense word pairings. A different set of shapes was made in the same way as those used in the visual-visual PAL condition. The nonsense words each contained three phonemes in CVC format. First, the experimenter asked the child to repeat the four non-words (e.g., vob, lep, dok, and haf) in order to make sure that he/she was able to pronounce them. The experimenter then held a set of four cards with different eight-point shapes. She showed the child one card at a time and said the associations twice. For example, ‘this shape goes with lep, [2-s interval], this shape goes with lep’. After all four pairs were introduced, the experimenter presented one card at a time and asked the child ‘which nonsense word goes with this shape?’ The experimenter recorded the child’s responses and feedback was given on each trial, in the same format as in the initial presentation, for example, “Do you remember? This shape goes with lep”. This procedure was also repeated five times, making a total of 20 learning trials/responses.

##### Visual – visual PAL

This condition of the task was assessed as a comparison to verbal-verbal and visual-verbal PAL. This test was included to ensure that the effects of PAL were not general across modalities. In this task, children were asked to learn which shapes went together. Two sets of cards with different eight-point shapes were used. Vanderplas and Garvin (1959), printed in black, and put onto cards. Each set contained four different shapes and had a different background color. First, one set of four shapes was laid out in a row in front of the child. The experimenter then placed each shape from the second set next to its pair from the first set. When the two shapes were placed adjacent to each other, the experimenter said ‘this shape goes with this shape’. The two shapes remained adjacent for 5 s, before the experimenter removed the card from the second set and placed the next card with the same procedure. After all four pairs were introduced, the experimenter shuffled the second set of cards and asked the child, one card at a time, ‘which shape goes with this shape?’ The child was asked to point to the correct match from the first set of shapes to be scored as correct. The experimenter recorded the child’s responses and provided feedback about which answer was correct. This procedure was repeated five times, making a total of 20 learning trials, which were all used as responses.

Visuospatial short-term memory was assessed in a *Matrix Pattern (MP)* span task (Wass et al., 2008). The procedure of the MP test is as follows: Patterns of filled cells are displayed in a 5 by 5 matrix on a computer screen. After presentation of any given pattern, the filled cells disappear and the child is then asked to click on the previously filled cells in an empty matrix. The level of difficulty increased from 1 to 8 filled cells. The task was discontinued when children made mistakes on at least 2 out of 3 patterns on two consecutive complexity levels. The children received span scores for the highest level of difficulty at which they correctly reproduced two out of three patterns. For example, if a child correctly reproduced two patterns with four filled cells, he/she received a span score of four. The maximum score in this test is 8.

##### Receptive vocabulary

The third edition of the Peabody Picture Vocabulary Test (PPVT-III; Dunn and Dunn, 2007) was used to assess receptive vocabulary. Participants were shown four pictures on each trial and they were asked to point to the picture that matched the word spoken by the experimenter. Testing began and ended according to test-specific basal and ceiling rules; testing ended after 8 errors were made in a set of 12 items.

Phonological short-term memory was assessed in the *Non-word Repetition test* (Sahlén et al., 1999). In this task, non-words of increasing syllable length were presented from the computer and the children were asked to orally repeat each non-word. The children’s responses were recorded on an external tape recorder and the recordings were subsequently used for scoring the accuracy of the responses. The repetition attempts were scored as percent consonants correctly reproduced (pcc).

Complex working memory was measured in the Sentence Completion and Recall task (Wass et al., 2008). The children were presented with sets of sentences with the last word missing and were required to complete and memorize the missing words, e.g., “Crocodiles are green. Tomatoes are ….”. Thereafter they were asked to repeat the words that they had previously filled in. The sentence sets consisted of two, three and four sentences. The score was the total number of correctly stored and reproduced words, with a maximum score of 18.

##### Expressive vocabulary

*Expressive vocabulary* was assessed using the *Boston Naming Test* (BNT; Kaplan et al., 1983). In this test, the children were required to name 60 drawn objects representing a range of nouns with varying frequency in language. Testing was performed in accordance with procedures from Tallberg (2005).

Word fluency was assessed with the *FAS letter fluency task* and the *Animal Fluency Task* (Benton and Hamshere, 1976). In the letter fluency task, which is a measure of phonological fluency, the children were required to say as many words as possible beginning with F, A, and S, respectively, within 1 min. In the *Animal Fluency Task*, which measures semantic fluency, the children were asked to say in 1 min as many words as possible belonging to the semantic category animals. In both of the tests, instruction and scoring procedures from Tallberg et al. (2011) were used.

Phonological skills were assessed in a phoneme deletion task (Magnusson and Nauclér, 1993). In this test, the children were asked to remove phoneme segments of spoken words, e.g., “Say summer without an ‘s”’. The maximum score in this test is 12.

### Procedure

All children were tested in connection to a regular follow-up appointment at the cochlear implant clinic, Karolinska University Hospital. They were assessed individually by a clinical speech language pathologist who was familiar to them. The tests were administered in two 1-h sessions, one per day on two consecutive days. All of the tests were presented in random order across both test sessions. The test instructions were given orally.

### Data Analysis

Raw scores were used as outcome measures in all analyses of the data except for Raven’s CPM for which percentile was used in the analyses.

In order to reduce the number of measures in the correlation analyses, a composite measure of word fluency (FAS and Animals) and expressive vocabulary (the Boston Naming test, BNT) was computed based on z-scores of these test measures. This composite measure, referred to as *word retrieval* was used in the correlation analyses. This choice of analysis was further guided by results from Riva et al. (2000) who studied word fluency in 160 children and found that only one significant factor underlies performance in the test measures Animals, FAS and BNT.

## Results

For the test measures where we had reference data from TH children, Table 2 shows the proportion of children with CI who performed within 1 SD, or higher, of the age mean for TH children. For some test measures where age norms were missing, the mean of previously collected age-matched TH children was used. For those test measures, the mean is based on *N* = 25 per age-group. We did not have access to norms or comparison data for the paired associate learning tests nor for the phoneme deletion test.

For some children, there are missing data in some of the tests. This is because the test times were set and in some cases we needed to prioritize among tests in order to keep the time limits.

All of the children except for one performed at ceiling on the word fluency measures (FAS and Animals) and more than two thirds of them performed within or above 1 SD of the mean of TH reference children on the expressive vocabulary test (Boston Naming). Twenty-five out of 32 children performed within 1 SD on the receptive vocabulary test (PPVT). The results were particularly low on the non-word repetition test which was used to measure phonological short-term memory. Only three out of the 22 children who completed the non-word repetition test performed within or above 1 SD of TH mean.

About 50 percent of the children performed within or above the 1 SD limit on the measures of complex and visual short-term memory capacity. All of the children had scores within or above 1 SD of TH mean on the reading fluency tests.

### Correlation Analyses

The relationships between orthographic learning, reading measures, and cognitive/linguistic skills were analyzed in second order Pearson partial correlation analyses with age and non-verbal IQ partialled out. These correlations are displayed in Table 3.

**Table 3:** Partial correlations between orthographic learning, reading and cognitive/linguistic skills.

	1	2	3	4	5	6	7	9	10	11	12	13	14
1. Orthographic Learning	1.000												
2. LäSt words	0.628^∗∗∗^	1.000											
3. LäSt non-words	0.729^∗∗∗^	0.858^∗∗∗^	1.000										
4. Orthographic Choices	0.462^∗^	0.609^∗∗∗^	0.617^∗∗∗^	1.000									
5. PPVT-III	0.636^∗∗∗^	0.313	0.393^∗^	0.221	1.000								
6. Word retrieval	0.823^∗∗∗^	0.736^∗∗∗^	0.691^∗∗∗^	0.406^∗^	0.595^∗∗^	1.000							
7. Phoneme deletion	0.651^∗∗∗^	0.591^∗∗^	0.611^∗∗^	0.364	0.460^∗^	0.647^∗∗∗^	1.000						
8. PAL verbal-verbal	0.660^∗∗∗^	0.269	0.406^∗^	0.310	0.521^∗∗^	0.548^∗∗^	0.265	1.000					
9. PAL visual-verbal	0.409^∗^	0.399^∗^	0.414^∗^	0.222	0.404^∗^	0.479^∗∗^	0.268	0.544^∗∗^	1.000				
10. PAL visual-visual	0.399^∗^	0.604^∗∗^	0.481^∗∗^	0.329	0.343	0.510^∗∗^	0.494^∗∗^	-0.003	0.287	1.000			
11. Matrix pattern	0.457^∗^	0.443^∗^	0.492^∗^	0.093	0.244	0.386	0.346	0.125	0.251	0.637^∗∗^	1.000		
12. Sentence completion and recall	0.338	0.418^∗^	0.272	-0.013	0.430^∗^	0.592^∗∗^	0.197	0.360	0.524^∗∗^	0.593^∗∗^	0.219	1.000	
13. Non-word repetition pcc	0.447^∗^	0.411	0.474^∗^	0.003	0.397	0.718^∗∗∗^	0.535^∗^	0.163	0.280	0.611^∗∗^	0.240	0.738^∗∗∗^	1.000


*Control for age and non-verbal IQ. ^∗^p < 0.05, ^∗∗^p < 0.01, ^∗∗∗^p < 0.001.*

Bonferroni correction for multiple comparisons was used in order to avoid Type-I errors. The significance level applied here was then α = 0.001. The correlations of interest were those between orthographic learning and word decoding fluency on the one hand and cognitive/linguistic measures on the other hand. The correlations that were significant at α < 0.001 are summarized and discussed below. However, for comparison, correlations that were significant at the conventional levels, α = 0.05 and α = 0.01 are marked with asterisks in Table 3 (^∗^ for α = 0.05 and ^∗∗^ for α = 0.01), but they are not discussed in the text.

Orthographic learning was significantly correlated with reading fluency (decoding of words and non-words), receptive vocabulary, word retrieval, phonological skills (phoneme deletion) and verbal-verbal PAL. Neither the correlation between orthographic learning and speech perception in quiet nor the correlations between orthographic learning and age at implantation of first or second CI, were significant after Bonferroni correction.

Word decoding fluency was strongly correlated with non-word decoding fluency, orthographic skills and word retrieval.

## Discussion

The aim of this study was to investigate the cognitive and linguistic correlates of orthographic learning in Swedish children with cochlear implants. A second aim was to compare the pattern of correlations between orthographic learning and cognitive/linguistic skills to the correlations between word decoding fluency and cognitive/linguistic skills in order to find out whether orthographic learning is dependent on the same underlying processes as word decoding.

The results showed strong associations between non-word decoding fluency and orthographic learning. Non-word decoding is a measure of childrens phonological decoding skills and this finding is thus in line with the self-teaching hypothesis (e.g., Share, 1995, 2004), which suggests that phonologogical decoding is necessary for orthographic learning. According to Share (1995, 2004) children memorize the orthographic representations of words as they read by means of phonological decoding.

The word decoding fluency test was also strongly correlated with orthographic learning. Phonological decoding may be the active mechanism in this relationship as well because children may use phonological decoding for reading words that they are not familiar with. When seeing a familiar word, on the other hand, the reader typically recognizes it immediately and performance in word decoding fluency tasks may thus be dependent on both phonological decoding skill and automatic word recognition. A relationship between orthographic learning and phonological decoding has recently been found in children with moderate – profound hearing loss (Wass et al., 2018) and this relationship has been demonstrated in numerous studies on TH children (e.g., Cunningham et al., 2002; De Jong et al., 2009; Wang et al., 2012, 2013). The current results thus indicate that phonological decoding is essential for the acquisition of orthographic representations, also for children with CI. This result is important in light of the findings from Nakeva von Mentzer et al. (2014) and Johnson and Goswami (2010) that children with a profound hearing loss who use CI have relatively more problems with phonological decoding (non-word decoding) as compared to orthographic word recognition (word decoding). That is, if children with CI are relatively poor at phonological decoding and this skill is important for orthographic learning, then specific intervention to improve phonological decoding should be warranted for all children with CI.

Four other cognitive and linguistic skills were strongly correlated with orthographic learning: receptive vocabulary, word retrieval, phonological skills (phoneme deletion), and verbal-verbal PAL.

The correlation between receptive vocabulary and orthographic learning is in line with previous results from TH children (Ricketts et al., 2007; Ouellette and Fraser, 2009) and children with moderate to profound hearing loss (Wass et al., 2018). According to Wegener et al. (2018) vocabulary may affect the process of orthographic learning when the child hears a spoken word for the first time, well before they see it in print. The authors suggest that children will then start to build up an anticipated representation of its orthographic form and that this anticipated representation then facilitates reading when the word is seen in print. That is, children with a more extensive vocabulary should be expected to have more anticipated orthographic representations for words that they have heard even if they have not yet seen them in print. Secondly, spoken vocabulary would be expected to play an important role in the process of orthographic learning by means of self-teaching as children have been suggested to utilize their receptive vocabulary in order to match unfamiliar written words to their spoken counterparts (c.f., Share, 1995; Ehri, 2014; Wegener et al., 2018). The relationship between orthographic learning and receptive vocabulary in children with CI is particularly important because vocabulary knowledge has been found to be poorer for this group of children than for typically hearing children (c.f., Geers et al., 2009). Receptive vocabulary may thus be an area of development for children with CI. That is, intervention to improve vocabulary knowledge may have the potential to improve orthographic learning and thereby reading skill in this group of children.

Word retrieval involves the quick retrieval of phonological and semantic information from long-term memory (e.g., Prigatano and Gray, 2008; Luo et al., 2010). A general interpretation of the relationship between orthographic learning and word retrieval may thus be that the ability to quickly retrieve language representations is important also for orthographic learning. In orthographic learning tasks, such as the one used in this study, the new representations are tested within a few minutes of the presentation phase. This means that the test items may well have been transferred to long-term memory although the memory trace is still relatively new and has not yet been strengthened by numerous instances of association and retrieval (c.f., Clay et al., 2007). In typical fluency tasks, on the other hand, the words to be retrieved are often consolidated memory representations of words that have been used and retrieved before. Irrespective of the level of consolidation of items to be retrieved, both orthographic learning and word retrieval taps the general ability to quickly retrieve words from memory.

Verbal-verbal PAL is the ability to associate two pieces of verbal information, in this case two nonsense words. Some previous studies have found associations between verbal-verbal PAL and reading measures in TH children (Hulme et al., 2007; Lervåg et al., 2009; Litt et al., 2013; Litt and Nation, 2014) but this skill has been considered secondary to the associations between cross-modal visual-verbal PAL and reading. The current result is in line with findings from a sample of Australian 9-year-old children with moderate-profound hearing loss (Wass et al., 2018). Their results showed significant correlations between orthographic learning and measures of verbal-verbal PAL and visual-verbal PAL.

Research on TH children suggests that cross-modal visual-verbal PAL is important for early reading because reading involves arbitrary association between visual and verbal information, for example when learning the names and sounds of letters (Lervåg et al., 2009) and when learning the pronunciations and spellings of irregular words (Messbauer and de Jong, 2003; Lervåg et al., 2009; Litt and Nation, 2014).

Litt et al. (2013) and Litt and Nation (2014), on the other hand, suggest that the ability to associate 2 pieces of verbal information rather than the ability to form cross-modal associations constitutes the core aspect of the relationship between PAL tasks and decoding. Litt and Nation (2014) point to their own findings and results from other studies (Hulme et al., 2007; Lervåg et al., 2009) demonstrating that verbal – verbal PAL is related to word decoding skill, and they claim that children, when reading a word for the first time, may come up with two different pronunciations of the word. One of the alternative pronunciations comes from their phonological decoding attempt, and may differ substantially from the correct pronunciation in particular if it is an irregular word (Litt et al., 2013). The other correct pronunciation may be provided by other people or may stem from inferences based on the children’s verbal vocabularies. The two alternative verbal representations are subsequently associated, that is, children learn that the word which sounds like “shoe” when decoded phonologically should be pronounced “show”. The association between verbal-verbal PAL and orthographic learning found in the current study is in line with the verbal account of PAL and suggests that the ability to associate 2 pieces of verbal information is important in the process of orthographic learning for children with CI.

Word decoding fluency was strongly correlated with three of the cognitive/linguistic skills: non-word decoding fluency, orthographic skill and word retrieval. The correlation between orthographic learning and non-word decoding fluency stresses the importance of phonological decoding in fluent reading for children with CI at a relatively early stage of reading acquisition (age 6–10). Previous findings from children with TH indicate that word recognition is strongly dependent on phonological decoding early in reading development (Castles and Nation, 2008; Castles et al., 2009) but that this relationship becomes less strong as children grow older and thus become able to recognize words instantly (Castles et al., 2009). The strong correlation between word decoding fluency and non-word decoding fluency may of course also be related to the fact that Swedish has relatively consistent grapheme-phoneme correspondences (van Daal and Wass, 2017). That is, graphemes do not vary substantially in the way that they are pronounced (Katz and Frost, 1992). Consequently, Swedish children, with or without a hearing loss, who are skilled at phonological decoding are thus likely to be able to read the vast majority of words by using a phonological decoding strategy already at a relatively early stage of reading development.

The association between non-word decoding and phoneme deletion was expected as phonological skills are well known to be related to phonological decoding, in particular for children early in reading development (e.g., Hulme et al., 2012).

In previous research, rapid naming has been found to be a strong predictor of word reading fluency (Savage and Frederickson, 2005; Georgiou et al., 2013) and the strong correlation between non-word decoding fluency and word retrieval in the current study is in line with those findings.

All in all orthographic learning and word decoding fluency were both strongly associated with phonological decoding fluency and word retrieval in this sample of children with CI. Orthographic learning was also correlated with receptive vocabulary, phonological skills and verbal-verbal PAL, whereas word decoding fluency was significantly correlated with existing orthographic knowledge. It is likely that the task of orthographic learning after only a brief exposure requires a broader range of skills than the reading task for this sample of children. Although the current study does not allow for conclusions about causality, the skills that were strongly correlated with orthographic learning in this study may indicate possible areas in which intervention programs may be applied in order to improve orthographic learning and thereby reading skill in children with CI. This is an important area for future research.

It should be noted that we did not find significant relations between orthographic learning and the demographic variables, age at implantation of first or second CI or hearing levels. The effects of these and other demographic variables such as etiology and educational setting should be further investigated in a larger sample of children with CI.

## Author Contributions

MW, UL, LA, EK, EÖ, and BL were involved in the planning, and writing phases of this study. UL, EÖ, and LA collected the data. MW prepared the draft. UL, LA, EK, BL, and EÖ contributed by reading and commenting on drafts of the manuscript.

## Conflict of Interest Statement

The authors declare that the research was conducted in the absence of any commercial or financial relationships that could be construed as a potential conflict of interest.
